# Diseases due to unhealthy environments: an updated estimate of the global burden of disease attributable to environmental determinants of health

**DOI:** 10.1093/pubmed/fdw085

**Published:** 2016-09-12

**Authors:** A. Prüss-Ustün, J. Wolf, C. Corvalán, T. Neville, R. Bos, M. Neira

**Affiliations:** 1 Department of Public Health, Environmental and Social Determinants of Health, World Health Organization , 1211Geneva, Switzerland; 2 Department of Epidemiology and Public Health, Swiss Tropical and Public Health Institute , 4051Basel, Switzerland; 3 University of Basel, 4003Basel, Switzerland; 4 Faculty of Health, University of Canberra, ACT2617, Australia; 5 Present address: World Health Organization, Geneva, Switzerland

**Keywords:** environment, morbidity and mortality, public health

## Abstract

**Background:**

The update of the global burden of disease attributable to the environment is presented. The study focuses on modifiable risks to show the potential health impact from environmental interventions.

**Methods:**

Systematic literature reviews on 133 diseases and injuries were performed. Comparative risk assessments were complemented by more limited epidemiological estimates, expert opinion and information on disease transmission pathways. Population attributable fractions were used to calculate global deaths and global disease burden from environmental risks.

**Results:**

Twenty-three percent (95% CI: 13–34%) of global deaths and 22% (95% CI: 13–32%) of global disability adjusted life years (DALYs) were attributable to environmental risks in 2012. Sixty-eight percent of deaths and 56% of DALYs could be estimated with comparative risk assessment methods. The global disease burden attributable to the environment is now dominated by noncommunicable diseases. Susceptible ages are children under five and adults between 50 and 75 years. Country level data are presented.

**Conclusions:**

Nearly a quarter of global disease burden could be prevented by reducing environmental risks. This analysis confirms that eliminating hazards and reducing environmental risks will greatly benefit our health, will contribute to attaining the recently agreed Sustainable Development Goals and will systematically require intersectoral collaboration to be successful.

## Introduction

Attribution of the burden of disease to environmental risks highlights the importance of environmental protection for people's health and can inform priority setting for targeted management of environmental determinants. Ten years ago the global burden of disease attributable to the environment was estimated for the first time in a comprehensive, systematic and transparent way.^1^ The study concluded that as much as 24% of disability adjusted life years (DALYs) and 23% of deaths were due to modifiable environmental risks.^1^

The health impacts of specific risk factors have traditionally been assessed separately.^2,3^ A comprehensive account of the consequences of unhealthy environments that are modifiable outlines the full potential of disease prevention that can be achieved by reconsidering the way we shape our environment. Since the last assessment 10 years ago,^1^ considerable more evidence has become available which justifies an updated assessment. We present here the methods and results of a new study which updates the previous analysis, by compiling the most recent synthesized and other key evidence on each disease and injury and their links to the environment.^4^ We present environmental burden of disease both in terms of environment-attributable mortality and DALYs, a weighted measure of death and disability.

The aim of the study is to quantify the links between disease or injury and environmental risks using CRAs and alternative methods and to derive an estimate of the environmental disease burden, overall, by region and country. For policy relevance, we deliberately focus on those risks which could be prevented or reduced by feasible interventions which modify the environment. The assessment was completed by a review of effective interventions for each of the investigated diseases.

## Methods

### Defining the environment in the context of public health

Environmental health has been defined as that part of public health that addresses all the physical, chemical and biological determinants of health external to a person, and all the related factors impacting behaviours.^5^ Included under environment for the purpose of this study are exposure to pollution and chemicals (e.g. air, water, soil, products), physical exposures (e.g. noise, radiation), the built environment (e.g. housing, land-use, infrastructure), other anthropogenic changes (e.g. climate change, vector breeding places), related behaviours and the work environment. Excluded are life style factors and behaviours which have no or only minor relations with the physical environment such as diet, tobacco or alcohol consumption, environments which cannot reasonably be modified (e.g. wetlands, pollen), or social conditions and unemployment. These risks are further detailed in Supplementary File (A1). The focus is placed on disease which can be prevented, either with almost immediate effect, or with longer term transformations.

### Systematic literature review

For each of the 133 disease and injury groups,^2^ we searched the literature systematically using Pubmed and Google Scholar for population health impacts from environmental risks and effects of interventions addressing those risks. The search strategy included a range of different MeSH (Medical Subject Headings) terms and keywords on each of the diseases or injuries, combined with terms for ‘environment’, ‘occupation’, relevant environmental risks and any of the occupational groups at risk, starting from the year 2004 until 2014. Older literature was taken from the earlier study^1^ and major projects of risk assessments were reviewed. Furthermore, the literature and data repositories were screened for documented and publicly available data and information on population health impacts, effects of interventions, exposure-response relationships, transmission pathways and causality. Global estimates of population impacts from environmental risks were completed with national or regional estimates, results of systematic reviews and meta-analyses on disease reduction from interventions or on environmental determinants; and finally by individual studies on interventions and environmental determinants. The focus on evidence of interventions underlines risk reductions that are already feasible, whereas other risk reductions may not yet be feasible or performed at large scale. Only risk factors with an established link of causality to health were further considered.

### Estimation of the population attributable fraction

The population attributable fraction (PAF) of a risk factor is the proportional reduction in population death or disease that would occur if exposure to this factor was removed or reduced to an achievable, alternative (or counterfactual) exposure distribution.^6^ To calculate the PAF of a risk factor to a disease, the following information is needed: (i) the exposure distribution to the risk factor within the population of interest, (ii) the relative risk (RR) linking each level of exposure to the specific disease or injury, and (iii) an alternative (counterfactual) exposure distribution to which environmental risks could be reduced. The counterfactual exposure distributions were based either on evidence from interventions, removal of pathways which have been eliminated elsewhere, or exposures achieved in some populations or areas.

According to the results of the systematic literature review (see above), four different approaches were used to estimate the fraction of diseases attributable to environmental risks in the following order of priority: (i) CRAs, which generally provide estimates based on the highest levels of evidence and most comprehensive data,^7–10^ (ii) estimates based on more limited exposure information and/or exposure-risk relationships, (iii) diseases with a transmission pathway dependent on specific modifiable environmental conditions were fully attributed to the environment (such as intestinal nematode infections which require contamination of the environment by human excreta), and (iv) expert surveys.

### Estimation of burden of disease attributable to the environment

In priority, we used systematic global estimates of population impacts from environmental risks (CRA type of assessments).^2,11–13^ These assessments are systematic evaluations of changes in population health resulting from modifying the population distribution of exposure from the current situation as compared to an alternative exposure, in combination with corresponding exposure-risk relationships. In these assessments, exposure is assessed for country populations as much as possible, the extrapolability of exposure-response relationship screened. CRA type of assessments are the method of choice and represent the highest level of evidence for environmental health conditions with a clear, established link between exposure and health outcome, such as exposure to air pollution or inadequate water and sanitation, chemicals or radiation. However, often available data is too limited to perform CRA type of assessments such as for insect vectors of diseases or rodent reservoirs of zoonoses which are more difficult to measure or which show a level of variation that is hard to translate in a disease burden, and alternative methods as specified below needed to be used.

Information on estimating disease burden from a combination of different risks is given in Supplementary File (A2).

When sufficient exposure distributions, or exposure-risk estimates or other important information was missing to perform CRA type of assessments, estimates based on more limited epidemiological data were performed, such as for HIV/AIDS, Hepatitis B, other sexually transmitted diseases, suicide and underweight.

Additional information can be found in the full WHO report on this work.^4^ For several diseases, approximate epidemiological estimations were also used to support expert opinion (e.g. unintentional injuries from fires).

Certain infectious diseases are solely transmitted through pathways which depend on specific modifiable environmental conditions, such as intestinal nematode infections which require contamination of the environment by human excreta. These diseases were fully attributed to the environment on the basis of their transmission pathway.

When estimates of population impacts from environmental risks were not available or could not be developed in the framework of this study, experts were asked to provide a best estimate of the fraction of the specific disease of the global population attributable to the reasonably modifiable environment, as well as the 95% confidence interval (CI). Experts were selected on the basis of their publications in the area of the disease or the relevant environmental risk factor. They were provided with abstracts of search results of the systematic reviews described earlier, as well as an initial estimate that was based on pooled estimates from the literature. Three or more experts were chosen for each disease or injury. More information on generating PAFs and confidence intervals from the experts' replies is given in Supplementary File (A3).

Where the body of evidence resulting from the updated literature review did not substantially differ or was unlikely to justify a change in experts' estimation of PAF, the results of the expert survey of the previous study^1^ were taken.

To calculate the fraction of disease attributable to a risk factor for any defined population, compiled or estimated PAFs were multiplied by the corresponding WHO disease statistics,^2^ by disease or injury, country, sex and age group, and for deaths and DALYs. Equations are listed in Supplementary File (A4).

### Compilation of main intervention areas

The evidence on effectiveness of interventions was further compiled by disease in order to summarize the main intervention areas.

## Results

**Fig.1:**
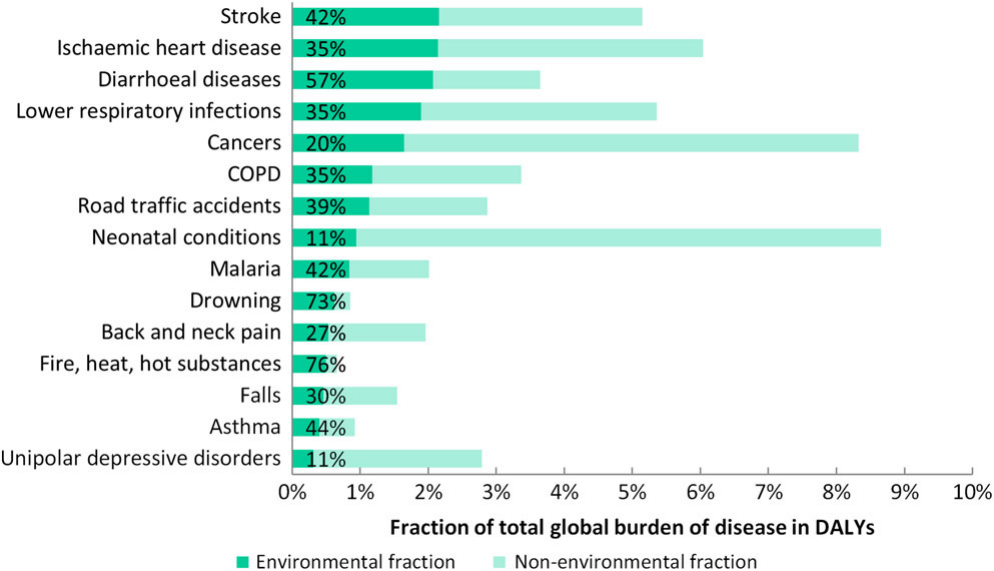
Environmental fraction of burden of selected diseases (percentages relate to the environmental share of the respective disease).

Results of environment-attributable deaths and disease burden, the attributable fractions, as well as the respective estimation method are listed in Table 1. The environmental fractions of the burden of selected diseases are shown in Fig. 1. Out of the 133 diseases or injuries, 101 had significant links with the environment, and 92 of them have been at least partially quantified. These 92 were grouped in 66 main disease and injury groups. Of these, global CRAs were available for 20 groups, of which 12 could be exclusively used for those diseases and eight needed to be completed by expert opinion. Eight diseases could be assessed (Table 1) on the basis of more limited epidemiological data, and four further disease PAFs were based on their transmission pathways. The PAFs of the remaining 31 diseases were fully estimated through expert surveys. More than 100 experts provided more than 250 quantitative replies. In terms of estimated environmental disease burden (in DALYs), as much as 56% could be estimated with CRA-type methods (of which 36% with a combination of risk factors), 40% were based on expert surveys (of which 8% in the 2015 round), 3% on estimations using more limited data, and 1% based on transmission pathways (Table 1).

**Table 1:** Global deaths, disease burden (in DALYs) and fractions attributable to the environment for 2012, and methods used

Disease	Deaths (in 2012)	DALYs (in 2012)	Attributable fraction (in DALYs) (95% CI)	Estimation method used
**Total**	12 624 495	596 412 171	22 (13–32)	
***Infectious and parasitic diseases***				
*Respiratory infections*				
Lower respiratory infections	566 361	51 752 605	35 (27–41)	a^e^
Upper respiratory infections and otitis	1190	989 751	14 (5–22)	d_2005_
*Diarrhoeal diseases*	845 810	56 606 914	57 (34–72)	a^f^
*Intestinal nematode infections*				
Ascariasis	3297	1 353 195	100	c
Trichuriasis	0	664 771	100	c
Hookworm disease	<10	3 211 578	100	c
*Parasitic and vector diseases*				
Malaria	258 702	23 074 449	42 (28–55)	d_2005_
Trachoma	0	298 711	100	c
Schistosomiasis	17 871	3 301 300	82 (71–92)	d_2015_
Chagas disease	4371	295 450	56 (28–80)	d_2005_
Lymphatic filariasis	<10	1 893 574	67 (39–89)	d_2005_
Onchocerciasis	0	59 827	10 (7–13)	d_2005_
Leishmaniasis	12 952	903 053	27 (9–40)	d_2005_
Dengue	27 249	1 369 867	95 (89–100)	d_2005_
*HIV/AIDS*^*#*^	137 985	7 780 321	10 (8–13)	b
*Sexually transmitted diseases excluding HIV/AIDS*^*#*^			8 (4–17)	
Syphilis	286	17 567	6 (3–14)	b
Chlamydia	108	115 567	8 (3–16)	b
Gonorrhoea	105	63 588	12 (7–25)	b
Trichomoniasis	0	6599	4 (2–6)	b
*Hepatitis B*	2828	111 446	2 (1–4)	b
*Tuberculosis*	166 687	7 688 971	18 (5–40)	(b), d_2005_
*Other infectious diseases*	160 418	11 463 450	27 (17–37)	d_2005_
***Neonatal and nutritional conditions***				
Neonatal conditions	270 087	25 819 566	11 (2–27)	d_2005_
Childhood underweight	27 291	2 834 186	15 (10–19)	b
***Noncommunicable diseases***				
Lung cancer	568 632	13 902 105	36 (17–52)	a^e^
Other cancers	1 097 144	31 047 781	16 (7–41)	(a), d_2005_
*Mental, behavioural and neurological disorders*				
Unipolar depressive disorders	536	8 473 707	12 (5–35)	d_2015_
Bipolar disorder	30	528 985	4 (0–9)	d_2015_
Schizophrenia	839	561 463	4 (1–9)	d_2015_
Alcohol use disorders	17 104	5 121 132	16 (6–38)	d_2015_
Drug use disorders	10 213	1 663 568	11 (2–36)	d_2015_
Anxiety disorders	13	5 479 365	20 (5–42)	d_2015_
Eating disorders	636	158 276	7 (0–20)	d_2015_
Pervasive developmental disorders	–	546 443	7 (0–26)	d_2015_
Childhood behavioural disorders	–	742 156	12 (3–36)	d_2015_
Idiopathic intellectual disability	106	193 742	6 (1–25)	d_2015_
Alzheimer‘s disease and other dementias	41 936	1 088 036	6 (1–13)	d_2015_
Parkinson‘s disease	8293	171 015	7 (2–14)	d_2015_
Epilepsy	30 031	3 023 792	15 (2–30)	d_2015_
Multiple sclerosis	1141	69 729	6 (1–22)	d_2015_
Migraine	<10	2 585 608	14 (2–36)	d_2015_
Non-migraine headache	–	310 613	17 (2–46)	d_2015_
Other mental, behavioural and neurological conditions	43 297	1 985 121	11 (2–24)	d_2015_
*Sense organ diseases*				
Cataracts	–	1 669 157	24 (14–33)	a^f^
Deafness	–	4 787 242	22 (19–25)	a^g^
*Cardiovascular diseases*				
Rheumatoid arthritis	10 928	934 393	17 (6–30)	a^g^
Hypertensive heart disease	93 652	2 146 830	9 (5–15)	a^g^
Ischaemic heart disease	2 273 811	58 561 915	35 (26–46)	a^e^
Stroke	2 476 553	58 985 984	42 (24–53)	a^e^
Other circulatory diseases	49 291	1 355 822	3 (1–5)	a^g^
*Respiratory diseases*				
Chronic obstructive pulmonary disease	1 193 589	32 280 160	35 (20–48)	a^e^
Asthma	169 449	11 055 150	44 (26–53)	(a), d_2005_
*Chronic kidney diseases*	27 143	759 826	3 (1–5)	a^g^
*Musculoskeletal diseases*				
Rheumatoid arthritis	6934	217–314	2 (1–4)	d_2005_
Osteoarthritis	829	3 606 529	20 (11–29)	d_2005_
Back and neck pain	158	14 627 733	27 (17–41)	a^g^, d_2015_
Other musculoskeletal diseases	20 666	4 961 741	15 (6–24)	d_2005_
*Congenital anomalies*	27 770	2 621 857	5 (1–10)	d_2005_
*Unintentional injuries*				
Road traffic accidents	497 079	31 000 887	39 (23–64)	(a), d_2005_
Unintentional Poisonings	137 339	7 824 627	73 (53–90)	(a), d_2005_
Falls	208 469	12 671 696	30 (15–58)	(a), d_2005_
Fires	199 776	13 665 389	76 (58–91)	(a), (b), d_2015_
Drownings	268 166	16 948 334	73 (43–94)	(a), d_2005_
Other unintentional injuries	393 136	23 133 586	43 (20–74)	(a), d_2005_
*Intentional injuries*				
Suicide	164 394	8 119 700	21 (13–30)	b
Interpersonal violence	81 730	5 101 921	16 (3–28)	d_2005_

HIV/AIDS = human immunodeficiency virus/acquired immunodeficiency syndrome; a: comparative risk assessment type, b: calculation based on limited epidemiological data, c: disease transmission pathway, d_2015_: expert survey 2015, d_2005_: expert survey 2005; () Estimates available, but completion by expert survey as main risk-factor disease pair not assessed. ^e^ Source: Combination of various risk factors developed for this analysis, WHO, based on references.^9,11–13^^f^ Source: WHO.^10,11^^g^ Source:^13^; see disease-specific sections and Technical Annex of full report for further information.

A description of the underlying evidence and region-specific results for each disease or injury are detailed in the report along with compiled effectiveness of environmental interventions. Based on a summary of the literature review on interventions, we report a mapping of diseases to main strategies for disease reduction through environmental improvements in Table 2, which are further detailed in the full report.^1^

**Table 2:** Main areas of strategies for disease reduction through environmental improvements

Disease or Injury^a^	Main areas
*Infectious and parasitic diseases*	
Respiratory infections	Household fuel use for cooking, heating and lighting, ambient air pollution, second-hand smoke, housing improvements (to prevent chilling, crowding).
Diarrhoeal diseases	Drinking water quality, improved sanitation facilities, recreational water quality, personal and community hygiene, animal excreta management, agricultural practices, climate change.
Intestinal nematode infections	Sanitation facilities and hygiene to prevent contamination of the environment with excreta, safe management of wastewater for irrigation.
Malaria	Environmental modification, including drainage, land levelling, filling depressions, pools and ponds, mosquito proof drinking water storage; environmental manipulation, including aquatic vegetation management, safe storage of domestic water, managing peri-domestic waste; reduced contact between humans and disease vectors screening of houses; livestock distribution.
Trachoma	Access to improved sanitation facilities; effective management of human waste; domestic water supplies, fly control, personal hygiene.
Schistosomiasis	Management of human waste, safe drinking water supply, improved irrigation infrastructure and safe irrigation and other agricultural practices; workers’ protection to avoid contact with contaminated water (such as wearing rubber boots).
Chagas disease	Management of peri-domestic areas (such as filling cracks in house walls, clearing areas around houses of wood stacks, maintaining goat corrals and chicken dens clean of organic debris).
Lymphatic filariasis	Modification of drainage and wastewater ponds, freshwater collection and irrigation schemes; impact depends on locally relevant disease vectors.
Onchocerciasis	Improved design and operation of water resources development projects (particularly dams).
Leishmaniasis	Housing improvements, such as eliminating soil and wall cracks, removal of organic material in the peri-domestic environment, workers’ personal protection.
Dengue	Management of water bodies around the house such as removing standing water from open water containers, urban infrastructure improvements, and solid waste management.
Japanese encephalitis	Irrigation management in rice-growing areas and distribution of farm animals (mainly pigs), personal protection methods.
HIV/AIDS and sexually transmitted diseases	Programmes to reduce occupational transmission among sex workers and migrant workers such as construction workers, seasonal agricultural labourers, truck drivers and sailors.
Hepatitis B and C	Occupational transmission among sex workers and migrant workers for hepatitis B;accidental needle-stick injuries in healthcare workers.
Tuberculosis	Exposure of miners and other occupational groups to airborne particles such as silica or coal dust, possibly exposure to household fuel combustion smoke and second-hand smoke. Managing setting-specific conditions, such as in prisons, hospitals and refugee camps.
*Neonatal and nutritional conditions*	
Neonatal conditions	Household air pollution from fuel combustion, mothers’ exposure to environmental tobacco smoke, poor water and sanitation in birth settings.
Childhood underweight	Provision of adequate water, sanitation and hygiene, adaptive management addressing climate change acting on food insecurity.
Cancers	Household air pollution from fuel combustion, ambient air pollution, second-hand smoke, ionizing radiation, ultraviolet radiation, exposure to chemicals, exposures at work and in other settings.
*Noncommunicable diseases*	
Neuropsychiatric disorders	Occupational stress has been linked to depression and anxiety; posttraumatic stress disorders to disasters such as floods, earthquakes, and fires, which could in part be prevented by environmental measures (e.g., floods by hydraulic infrastructure or land use patterns, or their mitigation of climate change, the impact of earthquakes and fires through more adequate buildings); forced resettlements in the context of development projects; drug use and alcohol disorder to the occupational environment such as working in the entertainment industry; epilepsy to occupational head trauma; Parkinson's disease to exposure to chemicals such as pesticides; intellectual disability to childhood exposure to lead and methylmercury; insomnia to noise and occupational stress; migraine to bright lights, poor air quality and odours. Exercise and physical activity fostered by supportive environments can reduce depression and anxiety.
Cataracts	Protection from ultraviolet radiation, reduction of household air pollution from combustion smoke.
Hearing loss	Managing occupational exposure to high noise levels.
Cardiovascular diseases	Reducing or eliminating indoor and outdoor air pollution, second-hand smoke, exposure to lead, stressful working conditions, shift work.
Chronic obstructive pulmonary disease	Reducing or eliminating household air pollution from combustion smoke, ambient air pollution, exposure to dusts in the workplace.
Asthma	Reducing or eliminating air pollution, second-hand smoke, exposure to indoor mould and dampness, occupational exposure to allergens.
Musculoskeletal diseases	Managing occupational stressors, such as heavy lifting, vibrations, prolonged sitting and poor work postures; need to carry large quantities of water over significant distances for domestic use.
Congenital anomalies	Mothers’ exposure to second-hand smoke, chemicals.
*Unintentional injuries*	
Road traffic accidents	Design of the roadways (e.g. sidewalks, bicycle lanes, restricted traffic, traffic-calming measures), land-use planning; traffic intensification in development areas with big infrastructure projects.
Unintentional poisonings	Safe handling and storage of chemicals, adequate product information, adequate choice of chemicals, workers’ protection (e.g. protective clothing).
Falls	Safety of housing and working environment.
Fires, heat and hot substances	Safety of cooking, lighting and heating equipment, in particular open fires, unsafe stoves or the use of candles or kerosene lamps, building fire codes, use of flammable materials in the home, safety of occupational environments and practices, climate change.
Drownings	Safety of water environments (community infrastructure, physical barriers, prevention and rescue services), public awareness, regulations (e.g. on transportation on waterways), workers’ safety measures, climate change-induced flood risks.
Other unintentional injuries	Protection from animal bites and contact with venomous plants, safety of mechanical equipment (including sports equipment, agricultural and industrial machinery), safety of off-road transportation, protection from exposure to ionizing radiation or electric currents.
*Intentional injuries*	
Self-harm	Access to toxic chemicals such as pesticides, access to firearms.
Interpersonal violence	Access to firearms, urban design (e.g. mobility, visibility), workers’ protection.
*Related risk factors*	
Physical inactivity	Workplace activity, prolonged sitting at the workplace, travel modes, transport infrastructure and land use patterns (walkability, urban density, land use diversity), availability of suitable parks and open spaces.
Obesity	Factors favouring physical activity.

^a^ Disease groups have been aggregated as compared to Table 1, as several disease subgroups have similar reduction strategies.

Environmental risks contributed 23% (95% CI = 13–34%) of the global burden measured in deaths, corresponding to 12.6 million deaths in 2012, and 22% (95% CI = 13–32%) in DALYs. In children under 5 years, as much as 26% of deaths and 25% of DALYs are attributable to the environment.

**Fig. 2:**
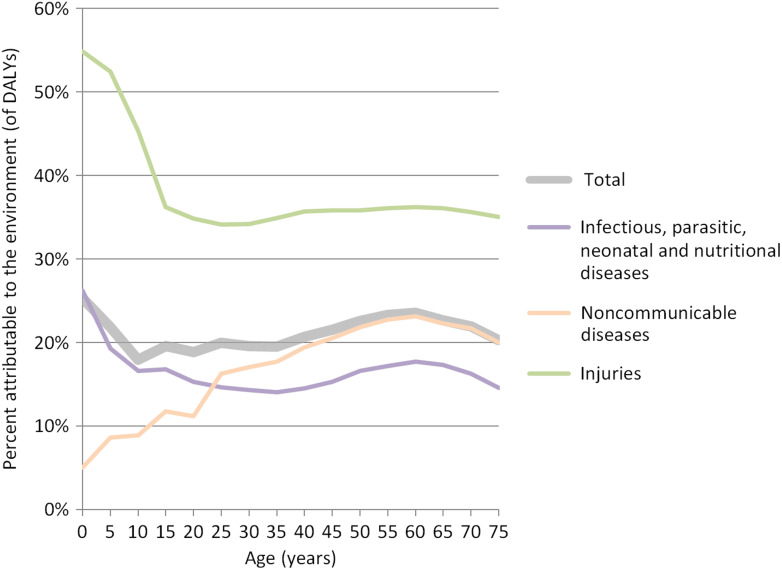
Environmental disease burden of overall; infectious, parasitic, neonatal and nutritional nutritional; noncommunicable diseases and injuries by age.

Global deaths attributable to the environment are dominated by 8.2 million deaths from noncommunicable diseases, followed by 2.5 million deaths related to infectious, parasitic, neonatal and nutritional diseases, and 2.0 million deaths from injuries. The difference is much less important in terms of disease burden, with 276, 202 and 118 million DALYs attributable to the environment in noncommunicable diseases; infectious, parasitic, neonatal and nutritional diseases; and injuries, respectively. Whereas there are significantly more deaths from noncommunicable diseases, infectious, parasitic, neonatal and nutritional diseases and injuries affect the young to a greater extent and therefore lead to relatively higher losses of DALYs relative to noncommunicable diseases (Fig. 2).

Figure 2 shows that overall disease burden attributable to the environment (thick grey line) peaks for the very young and for adults aged 50–75 years. These two age groups show important susceptibilities to environmental conditions. Children are mainly affected by communicable diseases. For the age group between 50 and 75 years the contributions of infectious diseases and injuries are still significant, while noncommunicable diseases, in particular cardiovascular diseases due to ambient and household air pollution, become very important. Box 1 highlights the shift from environmental disease burden from communicable to noncommunicable diseases between 2002 and 2012.

Box 1: **Trends of the environmental share of burden of disease by disease group.**

- Infectious, parasitic, neonatal and nutritional: PAF from 31% in 2002 to 20% in 2012
- Noncommunicable diseases: PAF from 17% in 2002 to 22% in 2012
- Injuries: PAF from 37% in 2002 to 38% in 2012
- Overall: PAF from 23.3% in 2002 to 22.7% in 2012

**Fig. 3:**
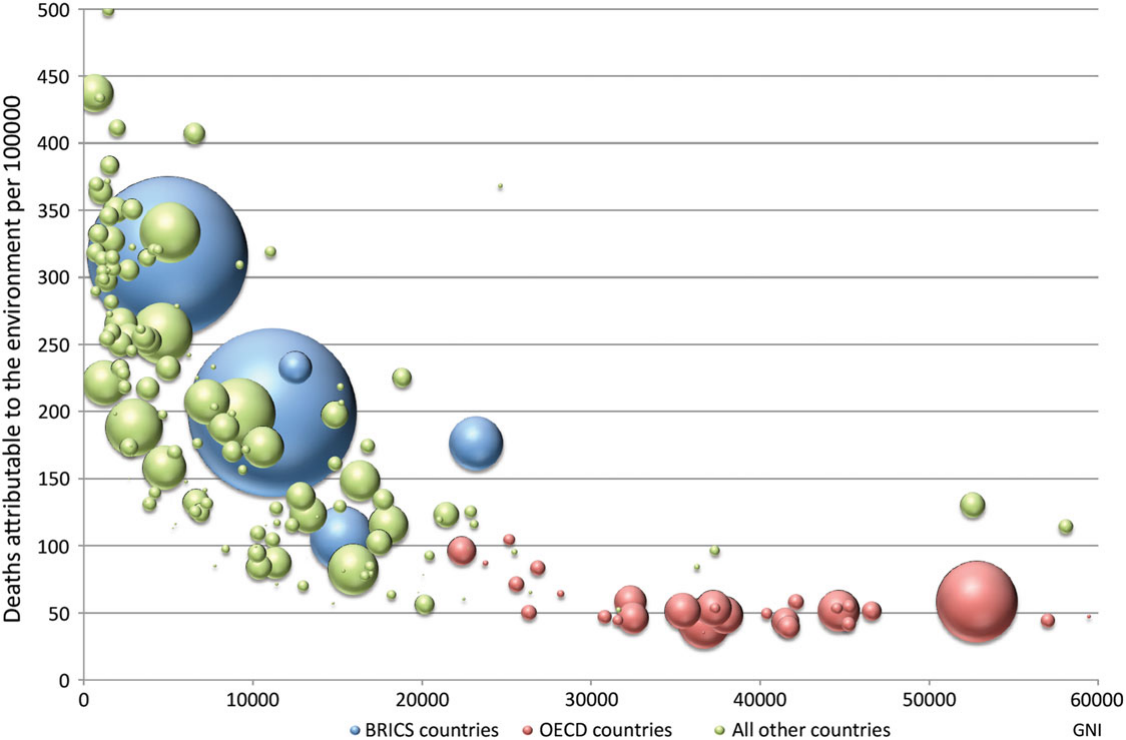
Environmental burden of disease (deaths per 100 000 population, *y*-axis) by gross national income per capita (*x*-axis); each bubble represents a country, bubble size represents population size; BRICS: Brazil, Russia, India, China, South Africa; OECD: Organisation for Economic Co-operation and Development.

Age-standardized deaths and DALYs by country are provided in Tables A1 and A2 of the Supplementary File. While the highest burden of environment-attributable disease is still in Sub-Saharan Africa and dominated by infectious, parasitic, neonatal and nutritional disease burden, the per capita deaths from noncommunicable diseases are now higher in most other regions of the world. Figure 3 shows environmentally related deaths per 100 000 population by gross national income (GNI). The size of the bubbles is proportional to country population. There is a reduction of deaths with increasing income up to a GNI of around 25 000. At larger incomes there is no difference in death rates, with most countries having around 50 deaths per 100 000.

## Discussion

### What is already known on the topic and what this study adds

Compared to our estimates for 2002, we see a major shift in the importance of environmental factors in noncommunicable disease aetiology. This is due to (i) the composition of the global disease burden which is now dominated by noncommunicable diseases,^14^ (ii) increased evidence on environmental determinants of noncommunicable diseases, and (iii) growing importance of environmental factors that contribute to noncommunicable diseases such as air pollution. As the world population continues to age rapidly, the trend of environmental risks predominantly affecting noncommunicable diseases is expected to become more pronounced.

One hundred and one out of 133 diseases and injuries were at least partially attributable to manageable environmental factors, as compared to 85 out of 102 in the previous study. In addition, the share of estimates based on the highest evidence level, i.e. using CRA type of approaches, has considerably increased and now reaches 56% (for DALYs), as compared to less than 10% in the previous study. In these high-evidence assessments, exposures are being assessed at country level or higher resolution, such as by age and gender to the extent possible and where appropriate, and the transferability of exposure-risk relationships to other population groups than where assessed are being verified or adjusted. This adds to the comprehensiveness and strength of evidence of the previous report.

Nevertheless, our numbers show that environmental factors continue to contribute to a large disease burden from communicable diseases in many low and middle income countries. In these countries, environmental risks leading to infectious diseases especially in children, such as household air pollution, unsafe drinking-water and poor sanitation and personal hygiene are still highly prevalent.^11,15^ Furthermore the burden from respiratory and intestinal infections in these countries remains high.^14^ At the same time they experience the double burden of communicable and noncommunicable diseases.

Our results differ from the Global Burden of Disease Study 2013 (GBD 2013)^8^ which attributed 12% of global DALYs and 16% of global mortality to environmental risks, mainly because we used a broader scope of the definition of environment and complementary methods of assessment. Those risks comprise unsafe water, sanitation and hygiene; air pollution (ambient particulate matter, ozone and household air pollution); second-hand smoke; lead and residential radon exposure; and occupational risks^8^ (NB: here we do not count burden attributable to physical inactivity/low physical activity as also for our analysis we did not quantify the environmental part of the burden from this risk factor). Our analysis covers a broader range of environmental risks adding noise (only included as occupational noise in GBD 2013); various chemicals; risks associated with poor housing, the recreational environment, water resource management, land use and the built environment; other community risks; radiation and climate change. Additionally, we consider more risk-factor disease links. Furthermore, GBD 2013 rated high blood pressure as most important risk factor, causing alone as much as 19% of global deaths and 8% of all DALYs.^8^ Some of this burden can however be attributed to environmental factors such as air pollution,^16,17^ arsenic^18^ and lead exposure,^19^ occupational risks^20^ and environmental noise.^21^

### Limitations of this study

A large part of this analysis is based on surveys of expert opinion and the uncertainties of such estimates are relatively large. However, experts were provided with the body of evidence that was identified during the systematic searches on the particular disease and its links to the respective environmental risks. We only updated this process when justified by a significant change in evidence. Further uncertainties relate to data limitations and assumptions made in e.g. CRA type of analyses.^8,11–13^ Also key exposures at younger ages, which may result in noncommunicable diseases at older ages could not be adequately captured in this study.

Certain diseases or environmental risk factors were not included in our analysis, either because there was insufficient evidence and therefore health effects were not quantifiable (e.g. changed, damaged or depleted ecosystems and exposure to endocrine disrupting substances), or because the risk factor(s) caused a relatively small disease burden, or is/are of regional significance but do not feature at a global scale. Environmental risks not readily modifiable, e.g. pollen, were not considered.

Additional conservative approaches have been chosen for this analysis as compared to the previous one in order to increase methodological rigour. For example, (a) only the main environmental risks were quantified where CRA estimates were available, and (b) the exposures of similar risks were combined before the estimation of health impacts. The environmental disease burden measured in DALYs between 2002 and 2012 is not directly comparable as some of the basic parameters as discounting and age-weighting for DALY estimation changed during this period.^22^ Using the same methods, the change would have been greater, as more deaths are now due to noncommunicable diseases, which tend to occur at older ages, and induce fewer years of life lost (and fewer DALYs).

We have not considered health impacts of social determinants.^23^ There is, however, a strong link between the conditions of people's daily lives and environmental risks to health. The lower people's socioeconomic status the more likely they are to be exposed to environmental risks, such as chemicals, air pollution and poor housing, water, sanitation and hygiene. Poor people and communities are therefore likely to benefit most from environmental interventions as they are disproportionally affected by adverse environments.^24^

### Policy implications

In principle, and given the methods and definitions chosen, the attributable burden here equates what can be prevented if the risks were removed. While we currently have solutions for reducing many of the prevailing risks, interventions that are affordable and that could completely eliminate certain risks such as ambient air pollution at a larger scale may require further development. Others, such as use of solid fuels, could be removed with almost immediate effect if the necessary means were made available. Yet for exposures which seem unavoidable in the short term, approaches are being considered which would require certain transformations in the way we currently produce and consume.

Important calls for action are coming from two main global platforms. One of them was created by the adoption of the SDGs in September 2015.^25^ It was significant that the Heads of State gathered at a Special Session of the UN General Assembly did not agree on another agenda or declaration, but made a pledge to ‘the transformation of our earth’. Full adherence to the obligations created by this pledge, even if only moral could result in important improvements on the reduction of environmental risks. The Supplementary File (A5, Table A3) gives further information on SDGs and their links with a healthy environment. The other is climate change. International efforts to reduce our carbon footprint (one such example is the recent Paris Agreement, the first global agreement to reduce climate change^26^) would lead to innovative interventions with positive ramifications to several key environmental factors, including to air pollution, water, chemicals, among others.

## Conclusions

This analysis, which confirms that reducing environmental exposures can greatly improve our health and is critical for attaining the SDGs, has been generated considering a large list of environmental risk factors and risk factor-disease links. For half of those links, CRA types of assessment were available basing the results on solid evidence.

In conclusion, our results convey good news as we included only those environmental exposures that are amenable to change, meaning that interventions exist for removing a large part of global disease burden. A prerequisite would be a stronger focus on primary prevention placing a healthy environment at the centre of such an effort. This is not a task for ministries of health alone. Tackling environmental risks requires intersectoral collaboration. After nearly 50 years of actively promoting this concept, whether referred to as intersectoral action, breaking down silos or the nexus approach, it remains elusive as ever. The statement ‘intersectoral collaboration: loved by all, funded by no-one’ points to obstacles, mainly vested interests, that have burdened this approach ever since it was included as part of the WHO/UNICEF Alma Ata Declaration on Primary Health Care in 1978. Environmental health, quintessentially intersectoral, has suffered most from this lack of progress. There remain a number of health sector-specific functions (monitoring, surveillance), but for the actual interventions the health sector will have to create the enabling environment for intersectoral action. Investing in environmental interventions pays off for governments; it reduces the transfer of hidden costs from other sectors to the health sector. This new report provides the evidence base for intersectoral action providing the evidence to systematically consider the integration of measures into all policy areas.

## Supplementary Material

Supplementary Data
