# Supplementary material for: Diseases due to unhealthy environments: an updated estimate of the global burden of disease attributable to environmental determinants of health
Source: J Public Health (Oxf). 2016 Sep 12;39(3):464–75. doi: 10.1093/pubmed/fdw085 (PMC5939845; doi:10.1093/pubmed/fdw085)
Supplement: Supplementary Data [file supplementary_file_prss_revised_clean.docx]

# Supplementary File

A1: Inclusions and exclusions to the definition of “environment” used in this study

Included environmental factors are the modifiable parts (or impacts) of:

- Pollution of air, water or soil by chemical or biological agents
- Ultraviolet (UV) ^[[1]](#footnote-1)^ and ionizing radiation
- Noise, electromagnetic fields
- Occupational risks
- Built environments, including housing; land-use patterns, roads
- Major infrastructural and engineering works such as roads, dams, railways, airports
- Man-made vector breeding places or breeding places catering to the specific ecological requirements of vectors, such as old tyres or water containers
- Agricultural methods, irrigation schemes
- Man-made climate change, ecosystem change
- Behaviour related to environmental factors, e.g. the availability of safe water for washing hands or physical activity fostered through improved urban design

Excluded factors are:

- Alcohol and tobacco consumption, drug abuse
- Diet (although it could be argued that food availability influences diet)
- The natural environments of vectors that cannot reasonably be modified (e.g. rivers, lakes, wetlands)
- Insecticide impregnated mosquito nets (for this study they are considered to be non-environmental interventions)
- Unemployment (provided that it is not related to environmental degradation, occupational disease, etc.)
- Natural biological agents, such as pollen
- Person-to-person transmission that cannot reasonably be prevented through environmental interventions, such as improving housing, introducing sanitary hygiene or making improvements in the occupational environment

A2: Combining estimates from environmental exposures

The disease burden is often the result of a combination of various risk factors. The PAFs of these factors may add up to more than 100% because most often these factors are not independent (2), meaning that disease burden could be potentially reduced or eliminated through tackling different risk factors. In this study, the aim was however to assess the combined effect of reducing environmental risks. In order to estimate the disease reduction that could be achieved when reducing all environmental risks to their alternative exposure distribution, the relevant risk factors for one disease were combined as follows:

- Exposures to the same pollutant but from different sources were considered as additive (e.g. air pollutants from ambient air and household air pollution and second-hand smoke), and the PAF for the combined exposures was estimated once for all combined risk factors.
- Exposures to different pollutants, or affecting very specific population subgroups (e.g. certain occupations), were estimated separately and combined using the product of complements for the PAFs:

$PAF=1-\prod_{r=1}^{R} (1-{PAF}_{r})$ (equation 1)

where PAF = population attributable fraction, and r = the individual risk factor.

For diseases with estimates of attributable burden from CRA methods for at least one major risk factor for the disease (mostly air pollution or water and sanitation, sometimes occupation – depending on the disease), no additional risk factor using other assessment methods was considered for estimating the overall PAF.

**A3: Generating PAFs and confidence intervals from expert survey**

Each expert reply was assumed to have a triangular probability distribution. The probability distributions of all expert replies for each disease were summed to determine a pooled probability distribution:

$P_{\mathrm{PAF}}$ = $\frac{\sum_{E=1}^{n} p_{PAF}}{n}$ (equation 2)

where PAF = population attributable fraction, P = resulting probability at population attributable fraction PAF, p = probability of individual expert at population attributable fraction PAF, E = experts, and n = number of experts.

The resulting mean PAF was defined as the overall best estimate. A new 95% confidence interval was defined as ranging from the 2.5 to the 97.5 percentiles of the pooled probability distribution of all the experts, calculated by generating 2,000 draws of each distribution. This method can lead to relatively large confidence intervals. Therefore, if an expert estimate did not overlap with any of the other experts’ estimates, this outliers’ best estimate was used to define the boundary of the pooled estimate, rather than the confidence interval from the expert.

**A4: Estimation of burden of disease attributable to the environment**

The following equations were used:

$AM=PAF \times M$ (equation 3)

and

$\mathrm{AB}\left( \mathrm{DALYs} \right)=PAF \times B\left( \mathrm{DALYs} \right)$ (equation 4)

Where AM = attributable mortality, PAF = population attributable fraction, M = mortality, AB (DALYs) = attributable burden in DALYs, and B (DALYs) = burden of disease in DALYs, for each disease or injury, country, sex and age group where relevant.

Table A1: Age-standardized deaths and DALYs per 100,000 capita attributable to the environment for infectious, parasitic, neonatal and nutritional diseases, noncommunicable diseases and injuries, by country and for the year 2012

| **Country** | **Age-standardized deaths / 100,000 capita** | | | | **Age-standardized DALYs / 100,000 capita** | | | |
| --- | --- | --- | --- | --- | --- | --- | --- | --- |
|  | **Infectious, parasitic, neonatal and nutritional** | **noncom­municable** | **injuries** | **total** | **infectious, parasitic, neonatal and nutritional** | **noncom­municable** | **injuries** | **total** |
| Afghanistan | 71 | 230 | 49 | 350 | 5,453 | 7,472 | 3,018 | 15,943 |
| Angola | 188 | 157 | 62 | 408 | 13,172 | 5,473 | 4,031 | 22,676 |
| Albania | 3 | 153 | 15 | 172 | 335 | 4,396 | 1,153 | 5,884 |
| Andorra | 0 | 38 | 4 | 43 | 61 | 1,985 | 392 | 2,439 |
| United Arab Emirates | 2 | 102 | 10 | 113 | 156 | 3,184 | 659 | 3,999 |
| Argentina | 4 | 62 | 18 | 84 | 369 | 2,598 | 1,078 | 4,044 |
| Armenia | 4 | 182 | 17 | 204 | 367 | 5,281 | 1,109 | 6,757 |
| Antigua and Barbuda | 2 | 66 | 10 | 79 | 183 | 2,894 | 946 | 4,022 |
| Australia | 1 | 33 | 5 | 39 | 69 | 1,756 | 419 | 2,245 |
| Austria | 0 | 48 | 6 | 55 | 66 | 2,173 | 455 | 2,694 |
| Azerbaijan | 10 | 140 | 11 | 161 | 902 | 4,349 | 952 | 6,203 |
| Burundi | 137 | 164 | 68 | 370 | 9,515 | 6,074 | 3,905 | 19,495 |
| Belgium | 1 | 49 | 8 | 58 | 76 | 2,139 | 532 | 2,746 |
| Benin | 96 | 176 | 42 | 314 | 7,159 | 5,721 | 2,325 | 15,205 |
| Burkina Faso | 108 | 184 | 53 | 344 | 7,908 | 5,990 | 2,865 | 16,763 |
| Bangladesh | 36 | 122 | 30 | 189 | 2,461 | 4,364 | 1,695 | 8,520 |
| Bulgaria | 2 | 117 | 10 | 129 | 239 | 3,789 | 820 | 4,847 |
| Bahrain | 3 | 71 | 10 | 83 | 201 | 2,566 | 614 | 3,382 |
| Bahamas | 3 | 45 | 12 | 60 | 264 | 2,074 | 979 | 3,318 |
| Bosnia and Herzegovina | 2 | 141 | 13 | 156 | 191 | 4,179 | 897 | 5,267 |
| Belarus | 2 | 138 | 34 | 173 | 168 | 4,644 | 1,719 | 6,531 |
| Belize | 8 | 52 | 25 | 85 | 685 | 2,302 | 1,824 | 4,811 |
| Bolivia (Plurinational State of) | 22 | 107 | 40 | 169 | 1,776 | 3,983 | 2,360 | 8,118 |
| Brazil | 10 | 70 | 25 | 105 | 631 | 3,060 | 1,441 | 5,132 |
| Barbados | 4 | 46 | 7 | 57 | 279 | 2,243 | 815 | 3,337 |
| Brunei Darussalam | 2 | 46 | 11 | 60 | 127 | 1,999 | 566 | 2,691 |
| Bhutan | 29 | 124 | 72 | 225 | 2,090 | 4,595 | 3,889 | 10,574 |
| Botswana | 85 | 96 | 37 | 218 | 4,990 | 3,677 | 1,958 | 10,625 |
| Central African Republic | 247 | 139 | 47 | 433 | 14,882 | 4,977 | 2,946 | 22,805 |
| Canada | 1 | 35 | 7 | 43 | 78 | 1,913 | 405 | 2,397 |
| Switzerland | 1 | 38 | 5 | 44 | 68 | 1,900 | 428 | 2,396 |
| Chile | 1 | 46 | 9 | 56 | 141 | 2,117 | 566 | 2,824 |
| China | 5 | 172 | 22 | 199 | 600 | 4,588 | 1,220 | 6,408 |
| Côte d'Ivoire | 134 | 163 | 53 | 350 | 9,714 | 5,801 | 3,232 | 18,746 |
| Cameroon | 126 | 133 | 46 | 305 | 8,659 | 4,858 | 2,721 | 16,239 |
| Democratic Republic of the Congo | 201 | 178 | 57 | 436 | 13,454 | 6,006 | 3,722 | 23,182 |
| Congo | 136 | 146 | 38 | 320 | 9,120 | 4,883 | 2,410 | 16,413 |
| Cook Islands | 7 | 43 | 25 | 75 | 1,054 | 1,855 | 1,325 | 4,235 |
| Colombia | 6 | 61 | 20 | 87 | 612 | 2,678 | 1,183 | 4,473 |
| Comoros | 97 | 147 | 61 | 305 | 5,938 | 5,138 | 2,949 | 14,025 |
| Cape Verde | 18 | 108 | 22 | 147 | 1,338 | 3,637 | 1,042 | 6,017 |
| Costa Rica | 3 | 51 | 16 | 69 | 405 | 2,392 | 826 | 3,623 |
| Cuba | 1 | 60 | 15 | 76 | 315 | 2,637 | 1,246 | 4,198 |
| Cyprus | 1 | 44 | 7 | 51 | 50 | 1,797 | 452 | 2,299 |
| Czech Republic | 1 | 73 | 9 | 82 | 80 | 2,552 | 647 | 3,279 |
| Germany | 1 | 46 | 4 | 51 | 77 | 2,104 | 399 | 2,580 |
| Djibouti | 87 | 101 | 45 | 232 | 4,968 | 3,716 | 2,281 | 10,966 |
| Dominica | 7 | 63 | 18 | 88 | 513 | 2,802 | 1,498 | 4,813 |
| Denmark | 1 | 47 | 5 | 52 | 77 | 2,107 | 395 | 2,579 |
| Dominican Republic | 8 | 71 | 25 | 105 | 755 | 2,755 | 1,950 | 5,460 |
| Algeria | 11 | 101 | 24 | 136 | 759 | 3,449 | 1,373 | 5,581 |
| Ecuador | 6 | 58 | 30 | 94 | 720 | 2,409 | 1,692 | 4,821 |
| Egypt | 9 | 150 | 14 | 173 | 872 | 4,607 | 960 | 6,440 |
| Eritrea | 93 | 150 | 55 | 298 | 5,048 | 5,199 | 2,534 | 12,781 |
| Spain | 1 | 41 | 4 | 46 | 77 | 1,750 | 389 | 2,217 |
| Estonia | 1 | 75 | 11 | 87 | 92 | 2,901 | 708 | 3,701 |
| Ethiopia | 86 | 94 | 41 | 221 | 5,646 | 3,790 | 2,187 | 11,622 |
| Finland | 0 | 41 | 8 | 50 | 58 | 1,989 | 537 | 2,584 |
| Fiji | 10 | 101 | 30 | 140 | 1,216 | 3,833 | 1,602 | 6,651 |
| France | 1 | 39 | 8 | 48 | 77 | 2,054 | 515 | 2,646 |
| Micronesia (Federated States of) | 29 | 73 | 31 | 133 | 1,680 | 2,978 | 1,649 | 6,307 |
| Gabon | 105 | 69 | 32 | 205 | 6,248 | 2,966 | 1,927 | 11,142 |
| United Kingdom | 1 | 47 | 5 | 53 | 80 | 2,293 | 446 | 2,819 |
| Georgia | 5 | 160 | 11 | 176 | 490 | 4,835 | 868 | 6,193 |
| Ghana | 78 | 144 | 32 | 255 | 5,955 | 4,898 | 1,883 | 12,735 |
| Guinea | 116 | 154 | 43 | 313 | 8,621 | 5,422 | 2,586 | 16,628 |
| Gambia | 92 | 139 | 41 | 272 | 6,045 | 4,923 | 2,337 | 13,305 |
| Guinea-Bissau | 148 | 174 | 49 | 371 | 10,515 | 5,620 | 2,769 | 18,903 |
| Equatorial Guinea | 150 | 162 | 56 | 367 | 9,613 | 5,500 | 3,423 | 18,536 |
| Greece | 1 | 63 | 7 | 70 | 73 | 2,429 | 566 | 3,068 |
| Grenada | 5 | 92 | 18 | 114 | 517 | 3,443 | 1,456 | 5,417 |
| Guatemala | 21 | 73 | 30 | 124 | 1,809 | 2,991 | 1,741 | 6,541 |
| Guyana | 29 | 163 | 50 | 242 | 1,874 | 5,305 | 3,057 | 10,236 |
| Honduras | 17 | 89 | 25 | 131 | 1,325 | 3,552 | 1,406 | 6,283 |
| Croatia | 1 | 82 | 9 | 91 | 83 | 2,981 | 621 | 3,686 |
| Haiti | 74 | 176 | 31 | 281 | 4,998 | 5,639 | 3,278 | 13,915 |
| Hungary | 1 | 113 | 10 | 124 | 110 | 3,968 | 697 | 4,776 |
| Indonesia | 25 | 152 | 21 | 198 | 1,667 | 4,601 | 1,211 | 7,479 |
| India | 62 | 207 | 46 | 315 | 3,527 | 6,297 | 2,294 | 12,119 |
| Ireland | 1 | 45 | 7 | 53 | 89 | 2,046 | 562 | 2,697 |
| Iran (Islamic Republic of) | 7 | 109 | 32 | 148 | 550 | 3,698 | 1,811 | 6,059 |
| Iraq | 13 | 144 | 40 | 198 | 1,039 | 4,292 | 2,011 | 7,342 |
| Iceland | 0 | 30 | 5 | 35 | 56 | 1,668 | 417 | 2,141 |
| Israel | 1 | 41 | 4 | 47 | 83 | 1,816 | 397 | 2,296 |
| Italy | 1 | 45 | 5 | 50 | 68 | 1,860 | 413 | 2,341 |
| Jamaica | 7 | 74 | 16 | 97 | 912 | 2,885 | 1,523 | 5,320 |
| Jordan | 5 | 101 | 22 | 127 | 499 | 3,355 | 1,251 | 5,105 |
| Japan | 1 | 33 | 8 | 41 | 62 | 1,616 | 432 | 2,110 |
| Kazakhstan | 6 | 185 | 34 | 224 | 518 | 5,698 | 1,931 | 8,147 |
| Kenya | 104 | 105 | 45 | 254 | 6,704 | 3,947 | 2,449 | 13,100 |
| Kyrgyzstan | 9 | 215 | 21 | 245 | 875 | 6,347 | 1,367 | 8,589 |
| Cambodia | 39 | 106 | 28 | 173 | 2,783 | 4,370 | 1,898 | 9,051 |
| Kiribati | 36 | 79 | 35 | 149 | 3,552 | 2,968 | 1,867 | 8,387 |
| Saint Kitts and Nevis | 3 | 47 | 15 | 65 | 293 | 1,849 | 1,206 | 3,348 |
| Republic of Korea | 2 | 49 | 7 | 58 | 108 | 1,968 | 385 | 2,461 |
| Kuwait | 5 | 70 | 9 | 84 | 234 | 2,561 | 680 | 3,476 |
| Lao People's Democratic Republic | 101 | 187 | 32 | 321 | 6,857 | 5,787 | 1,879 | 14,524 |
| Lebanon | 2 | 69 | 13 | 85 | 274 | 2,389 | 727 | 3,390 |
| Liberia | 96 | 157 | 36 | 289 | 6,955 | 5,281 | 1,973 | 14,210 |
| Libya | 6 | 96 | 23 | 126 | 454 | 3,275 | 1,224 | 4,954 |
| Saint Lucia | 4 | 77 | 18 | 99 | 420 | 3,076 | 2,183 | 5,678 |
| Sri Lanka | 8 | 128 | 34 | 169 | 567 | 4,089 | 1,609 | 6,265 |
| Lesotho | 155 | 109 | 58 | 323 | 8,883 | 4,234 | 3,230 | 16,347 |
| Lithuania | 2 | 95 | 19 | 116 | 145 | 3,394 | 1,093 | 4,632 |
| Luxembourg | 1 | 39 | 7 | 47 | 59 | 1,943 | 480 | 2,481 |
| Latvia | 1 | 104 | 14 | 120 | 133 | 3,516 | 881 | 4,530 |
| Morocco | 13 | 99 | 20 | 133 | 994 | 3,563 | 1,350 | 5,907 |
| Monaco | 1 | 26 | 7 | 34 | 78 | 1,362 | 539 | 1,979 |
| Republic of Moldova | 5 | 165 | 27 | 196 | 429 | 4,845 | 1,508 | 6,781 |
| Madagascar | 82 | 176 | 39 | 296 | 5,667 | 5,988 | 2,168 | 13,824 |
| Maldives | 5 | 51 | 15 | 72 | 423 | 2,167 | 805 | 3,394 |
| Mexico | 5 | 57 | 20 | 82 | 580 | 2,212 | 1,170 | 3,962 |
| Marshall Islands | 44 | 66 | 19 | 129 | 3,487 | 2,726 | 1,163 | 7,376 |
| The former Yugoslav Republic of Macedonia | 2 | 108 | 7 | 117 | 191 | 3,678 | 628 | 4,498 |
| Mali | 129 | 202 | 52 | 383 | 9,441 | 6,189 | 2,844 | 18,473 |
| Malta | 0 | 61 | 4 | 65 | 74 | 2,352 | 396 | 2,822 |
| Myanmar | 47 | 191 | 39 | 277 | 3,309 | 5,799 | 2,147 | 11,255 |
| Montenegro | 1 | 111 | 9 | 121 | 168 | 3,826 | 720 | 4,714 |
| Mongolia | 13 | 265 | 31 | 309 | 1,134 | 7,584 | 1,947 | 10,665 |
| Mozambique | 171 | 114 | 78 | 363 | 10,903 | 4,408 | 3,872 | 19,183 |
| Mauritania | 110 | 115 | 36 | 261 | 6,672 | 4,020 | 1,914 | 12,607 |
| Mauritius | 6 | 55 | 18 | 79 | 476 | 2,390 | 1,095 | 3,961 |
| Malawi | 124 | 150 | 44 | 318 | 7,756 | 4,937 | 2,111 | 14,803 |
| Malaysia | 6 | 90 | 27 | 123 | 1,053 | 3,096 | 1,356 | 5,504 |
| Namibia | 52 | 114 | 31 | 197 | 3,186 | 3,916 | 1,694 | 8,795 |
| Niger | 141 | 147 | 44 | 333 | 9,353 | 5,047 | 2,474 | 16,873 |
| Nigeria | 140 | 131 | 62 | 333 | 10,441 | 4,714 | 3,644 | 18,799 |
| Nicaragua | 11 | 105 | 23 | 139 | 1,009 | 3,941 | 1,305 | 6,256 |
| Niue | 12 | 49 | 31 | 93 | 1,409 | 1,927 | 1,682 | 5,018 |
| Netherlands | 1 | 46 | 4 | 51 | 70 | 2,036 | 372 | 2,477 |
| Norway | 1 | 35 | 6 | 42 | 66 | 2,018 | 409 | 2,493 |
| Nepal | 41 | 172 | 37 | 251 | 2,608 | 5,556 | 1,964 | 10,129 |
| Nauru | 11 | 30 | 15 | 56 | 1,420 | 1,427 | 855 | 3,703 |
| New Zealand | 1 | 37 | 6 | 44 | 73 | 1,935 | 496 | 2,504 |
| Oman | 4 | 73 | 19 | 96 | 269 | 2,564 | 1,024 | 3,857 |
| Pakistan | 62 | 155 | 40 | 258 | 4,320 | 4,798 | 2,267 | 11,385 |
| Panama | 12 | 46 | 19 | 77 | 795 | 2,294 | 1,144 | 4,233 |
| Peru | 6 | 59 | 19 | 85 | 626 | 2,695 | 1,118 | 4,439 |
| Philippines | 27 | 160 | 19 | 206 | 2,032 | 5,541 | 1,236 | 8,809 |
| Palau | 9 | 53 | 29 | 92 | 1,096 | 1,974 | 1,466 | 4,536 |
| Papua New Guinea | 68 | 102 | 48 | 218 | 4,547 | 4,098 | 2,503 | 11,148 |
| Poland | 1 | 84 | 11 | 95 | 91 | 3,114 | 773 | 3,978 |
| Democratic People's Republic of Korea | 14 | 260 | 37 | 310 | 1,208 | 6,900 | 2,014 | 10,122 |
| Portugal | 1 | 44 | 5 | 50 | 79 | 2,193 | 451 | 2,723 |
| Paraguay | 8 | 93 | 24 | 125 | 773 | 3,612 | 1,391 | 5,775 |
| Qatar | 2 | 59 | 11 | 73 | 178 | 2,426 | 658 | 3,262 |
| Romania | 4 | 118 | 12 | 135 | 394 | 3,921 | 942 | 5,257 |
| Russian Federation | 3 | 143 | 30 | 176 | 259 | 4,819 | 1,640 | 6,717 |
| Rwanda | 77 | 135 | 42 | 254 | 5,256 | 4,971 | 2,300 | 12,527 |
| Saudi Arabia | 4 | 110 | 16 | 130 | 242 | 3,363 | 908 | 4,514 |
| Sudan | 86 | 113 | 53 | 252 | 5,877 | 4,353 | 2,804 | 13,034 |
| Senegal | 96 | 98 | 38 | 231 | 5,342 | 3,654 | 1,834 | 10,829 |
| Singapore | 1 | 43 | 3 | 47 | 87 | 1,605 | 208 | 1,900 |
| Solomon Islands | 36 | 128 | 34 | 198 | 2,253 | 4,547 | 1,803 | 8,604 |
| Sierra Leone | 223 | 210 | 66 | 499 | 15,667 | 7,038 | 3,940 | 26,645 |
| El Salvador | 6 | 81 | 43 | 130 | 737 | 3,339 | 2,263 | 6,338 |
| San Marino | 0 | 22 | 6 | 29 | 64 | 1,207 | 490 | 1,761 |
| Somalia | 195 | 117 | 57 | 369 | 12,240 | 4,610 | 3,293 | 20,143 |
| Serbia | 2 | 106 | 7 | 115 | 163 | 3,869 | 671 | 4,703 |
| South Sudan | 135 | 115 | 56 | 306 | 8,884 | 3,950 | 3,098 | 15,932 |
| Sao Tome and Principe | 54 | 82 | 31 | 166 | 3,512 | 3,373 | 1,780 | 8,665 |
| Suriname | 9 | 50 | 22 | 81 | 642 | 2,418 | 1,819 | 4,879 |
| Slovakia | 1 | 93 | 10 | 104 | 98 | 3,182 | 729 | 4,010 |
| Slovenia | 0 | 53 | 10 | 63 | 77 | 2,305 | 620 | 3,002 |
| Sweden | 1 | 35 | 5 | 41 | 64 | 1,892 | 411 | 2,367 |
| Swaziland | 123 | 107 | 48 | 278 | 7,796 | 4,057 | 2,750 | 14,603 |
| Seychelles | 14 | 50 | 34 | 98 | 1,034 | 2,189 | 1,421 | 4,644 |
| Syrian Arab Republic | 5 | 121 | 14 | 140 | 523 | 3,698 | 901 | 5,123 |
| Chad | 204 | 156 | 51 | 412 | 13,426 | 5,536 | 2,939 | 21,900 |
| Togo | 116 | 148 | 40 | 305 | 8,359 | 5,284 | 2,419 | 16,062 |
| Thailand | 8 | 85 | 30 | 124 | 655 | 3,175 | 1,560 | 5,389 |
| Tajikistan | 22 | 187 | 19 | 228 | 1,887 | 5,671 | 1,384 | 8,942 |
| Turkmenistan | 18 | 271 | 30 | 319 | 1,487 | 8,720 | 2,023 | 12,230 |
| Timor-Leste | 58 | 145 | 30 | 233 | 3,863 | 4,774 | 1,812 | 10,450 |
| Tonga | 10 | 82 | 21 | 114 | 697 | 3,312 | 1,137 | 5,147 |
| Trinidad and Tobago | 4 | 70 | 21 | 94 | 274 | 2,816 | 1,437 | 4,528 |
| Tunisia | 4 | 88 | 17 | 109 | 405 | 3,145 | 1,045 | 4,595 |
| Turkey | 3 | 102 | 10 | 115 | 323 | 3,648 | 798 | 4,769 |
| Tuvalu | 22 | 74 | 38 | 134 | 1,773 | 2,706 | 1,898 | 6,377 |
| United Republic of Tanzania | 107 | 102 | 56 | 264 | 6,634 | 4,131 | 2,837 | 13,602 |
| Uganda | 117 | 139 | 71 | 327 | 7,558 | 5,095 | 3,638 | 16,290 |
| Ukraine | 4 | 159 | 24 | 187 | 350 | 4,839 | 1,395 | 6,583 |
| Uruguay | 1 | 50 | 12 | 64 | 149 | 2,272 | 701 | 3,122 |
| United States of America | 1 | 46 | 11 | 57 | 95 | 2,190 | 611 | 2,896 |
| Uzbekistan | 11 | 205 | 16 | 232 | 995 | 6,224 | 1,220 | 8,438 |
| Saint Vincent and the Grenadines | 7 | 82 | 19 | 108 | 642 | 3,111 | 1,584 | 5,337 |
| Venezuela (Bolivarian Republic of) | 8 | 63 | 31 | 101 | 676 | 2,748 | 1,896 | 5,320 |
| Viet Nam | 13 | 119 | 26 | 158 | 1,326 | 3,997 | 1,441 | 6,764 |
| Vanuatu | 23 | 124 | 27 | 173 | 1,468 | 4,257 | 1,275 | 7,001 |
| Samoa | 11 | 81 | 23 | 116 | 784 | 3,162 | 1,187 | 5,132 |
| Yemen | 33 | 151 | 33 | 216 | 2,810 | 5,158 | 2,122 | 10,091 |
| South Africa | 88 | 100 | 44 | 232 | 5,131 | 3,607 | 2,261 | 10,998 |
| Zambia | 125 | 124 | 65 | 315 | 8,538 | 4,476 | 3,227 | 16,241 |
| Zimbabwe | 107 | 118 | 34 | 259 | 7,151 | 4,246 | 2,201 | 13,598 |
| **Gobal** | **35** | **137** | **29** | **201** | **2,340** | **4,368** | **1,619** | **8,326** |

values in this table are suitable for inter-county comparisons

Table A2: Deaths and DALYs (absolute numbers) attributable to the environment for infectious, parasitic, neonatal and nutritional diseases, noncommunicable diseases and injuries, by country and for the year 2012

| **Country** | **Deaths** | | | | | **DALYs** | | | | |
| --- | --- | --- | --- | --- | --- | --- | --- | --- | --- | --- |
|  | **Infectious, parasitic, neonatal and nutritional** | **noncom­municable** | **injuries** | **total** | **PAF** | **infectious, parasitic, neonatal and nutritional** | **noncom­municable** | **injuries** | **total** | **PAF** |
| Afghanistan | 29,571 | 22,490 | 13,086 | 65,147 | 0.26 | 2,676,657 | 1,071,876 | 986,768 | 4,735,300 | 0.24 |
| Angola | 53,081 | 11,952 | 13,502 | 78,536 | 0.26 | 4,580,128 | 615,893 | 1,033,990 | 6,230,011 | 0.27 |
| Albania | 78 | 6,174 | 514 | 6,767 | 0.23 | 8,078 | 165,279 | 36,697 | 210,054 | 0.20 |
| Andorra | 0 | 62 | 6 | 68 | 0.11 | 45 | 2,142 | 408 | 2,594 | 0.12 |
| United Arab Emirates | 82 | 1,303 | 753 | 2,138 | 0.22 | 11,338 | 140,714 | 70,702 | 222,754 | 0.16 |
| Argentina | 1,715 | 30,920 | 8,316 | 40,951 | 0.13 | 150,200 | 1,146,157 | 450,770 | 1,747,127 | 0.14 |
| Armenia | 124 | 7,035 | 549 | 7,707 | 0.21 | 9,813 | 183,201 | 34,445 | 227,459 | 0.19 |
| Antigua and Barbuda | 2 | 62 | 10 | 74 | 0.12 | 161 | 2,613 | 840 | 3,614 | 0.13 |
| Australia | 224 | 13,063 | 1,774 | 15,061 | 0.10 | 14,761 | 504,791 | 112,558 | 632,110 | 0.12 |
| Austria | 50 | 8,605 | 897 | 9,552 | 0.12 | 4,827 | 268,269 | 50,785 | 323,881 | 0.13 |
| Azerbaijan | 826 | 10,221 | 993 | 12,040 | 0.21 | 78,232 | 357,489 | 86,907 | 522,628 | 0.18 |
| Burundi | 17,019 | 6,252 | 6,309 | 29,580 | 0.26 | 1,470,816 | 323,361 | 458,983 | 2,253,160 | 0.27 |
| Belgium | 196 | 11,560 | 1,529 | 13,286 | 0.12 | 8,581 | 347,876 | 74,980 | 431,436 | 0.13 |
| Benin | 11,815 | 6,922 | 3,579 | 22,316 | 0.25 | 1,063,310 | 315,558 | 254,648 | 1,633,515 | 0.25 |
| Burkina Faso | 24,035 | 10,480 | 7,291 | 41,806 | 0.26 | 2,147,711 | 490,618 | 527,192 | 3,165,520 | 0.26 |
| Bangladesh | 48,257 | 113,704 | 39,573 | 201,534 | 0.23 | 3,844,616 | 4,949,517 | 2,552,303 | 11,346,436 | 0.22 |
| Bulgaria | 120 | 17,511 | 923 | 18,555 | 0.17 | 11,865 | 450,772 | 70,555 | 533,192 | 0.17 |
| Bahrain | 21 | 327 | 94 | 442 | 0.16 | 2,269 | 24,201 | 8,270 | 34,740 | 0.14 |
| Bahamas | 10 | 148 | 43 | 201 | 0.09 | 888 | 7,687 | 3,639 | 12,215 | 0.11 |
| Bosnia and Herzegovina | 66 | 8,977 | 638 | 9,681 | 0.27 | 5,417 | 235,729 | 40,063 | 281,209 | 0.23 |
| Belarus | 154 | 19,848 | 3,807 | 23,809 | 0.19 | 14,412 | 581,509 | 182,907 | 778,828 | 0.19 |
| Belize | 22 | 91 | 65 | 177 | 0.13 | 2,270 | 5,560 | 5,245 | 13,075 | 0.16 |
| Bolivia (Plurinational State of) | 2,442 | 6,942 | 3,837 | 13,222 | 0.18 | 220,263 | 312,512 | 254,810 | 787,586 | 0.19 |
| Brazil | 17,793 | 128,381 | 49,568 | 195,742 | 0.15 | 1,173,341 | 5,901,830 | 2,893,254 | 9,968,425 | 0.16 |
| Barbados | 10 | 170 | 23 | 204 | 0.11 | 695 | 7,369 | 2,539 | 10,603 | 0.12 |
| Brunei Darussalam | 6 | 125 | 37 | 167 | 0.12 | 468 | 7,268 | 2,172 | 9,909 | 0.12 |
| Bhutan | 198 | 571 | 490 | 1,259 | 0.26 | 15,813 | 25,321 | 29,127 | 70,261 | 0.25 |
| Botswana | 1,352 | 944 | 610 | 2,905 | 0.18 | 94,149 | 49,154 | 39,191 | 182,494 | 0.18 |
| Central African Republic | 11,093 | 3,021 | 2,025 | 16,138 | 0.25 | 833,592 | 139,051 | 145,114 | 1,117,757 | 0.25 |
| Canada | 503 | 22,225 | 3,615 | 26,343 | 0.11 | 25,584 | 880,118 | 163,207 | 1,068,909 | 0.12 |
| Switzerland | 79 | 6,387 | 760 | 7,226 | 0.12 | 4,832 | 215,977 | 44,928 | 265,737 | 0.13 |
| Chile | 262 | 9,337 | 1,683 | 11,282 | 0.12 | 23,094 | 403,140 | 102,867 | 529,101 | 0.13 |
| China | 54,470 | 2,607,982 | 324,232 | 2,986,684 | 0.30 | 6,804,206 | 71,784,861 | 17,379,151 | 95,968,218 | 0.26 |
| Côte d'Ivoire | 30,828 | 14,589 | 10,077 | 55,493 | 0.22 | 2,626,871 | 708,396 | 730,661 | 4,065,928 | 0.23 |
| Cameroon | 30,628 | 12,809 | 9,489 | 52,926 | 0.22 | 2,577,475 | 623,933 | 690,090 | 3,891,498 | 0.23 |
| Democratic Republic of the Congo | 163,548 | 45,269 | 39,015 | 247,832 | 0.27 | 13,756,082 | 2,149,741 | 2,961,781 | 18,867,604 | 0.27 |
| Congo | 6,659 | 2,840 | 1,579 | 11,078 | 0.25 | 551,214 | 125,713 | 116,248 | 793,175 | 0.25 |
| Cook Islands | 1 | 7 | 5 | 13 | 0.13 | 218 | 337 | 258 | 814 | 0.15 |
| Colombia | 2,534 | 22,795 | 8,967 | 34,297 | 0.17 | 298,949 | 1,142,670 | 559,194 | 2,000,813 | 0.16 |
| Comoros | 732 | 447 | 328 | 1,508 | 0.25 | 59,374 | 21,294 | 21,018 | 101,686 | 0.25 |
| Cape Verde | 73 | 370 | 91 | 533 | 0.21 | 6,513 | 13,411 | 4,742 | 24,665 | 0.18 |
| Costa Rica | 116 | 2,213 | 770 | 3,099 | 0.15 | 18,297 | 111,024 | 41,082 | 170,403 | 0.16 |
| Cuba | 176 | 10,323 | 2,432 | 12,931 | 0.15 | 31,615 | 377,445 | 167,593 | 576,653 | 0.16 |
| Cyprus | 9 | 716 | 93 | 818 | 0.13 | 528 | 24,782 | 5,809 | 31,120 | 0.14 |
| Czech Republic | 154 | 14,306 | 1,342 | 15,802 | 0.15 | 8,196 | 405,823 | 84,677 | 498,696 | 0.15 |
| Germany | 1,630 | 90,544 | 6,697 | 98,870 | 0.11 | 62,648 | 2,742,226 | 456,105 | 3,260,979 | 0.13 |
| Djibouti | 643 | 450 | 316 | 1,409 | 0.18 | 47,797 | 22,394 | 20,197 | 90,388 | 0.17 |
| Dominica | 6 | 48 | 13 | 67 | 0.13 | 366 | 2,041 | 1,072 | 3,479 | 0.15 |
| Denmark | 92 | 5,098 | 424 | 5,615 | 0.10 | 4,248 | 167,416 | 28,563 | 200,227 | 0.12 |
| Dominican Republic | 897 | 6,020 | 2,444 | 9,361 | 0.19 | 86,274 | 245,383 | 192,003 | 523,659 | 0.18 |
| Algeria | 3,806 | 24,914 | 7,999 | 36,718 | 0.17 | 319,961 | 1,052,675 | 520,484 | 1,893,120 | 0.16 |
| Ecuador | 973 | 7,522 | 4,330 | 12,825 | 0.16 | 118,779 | 330,403 | 260,272 | 709,454 | 0.16 |
| Egypt | 7,283 | 84,308 | 10,215 | 101,806 | 0.19 | 764,086 | 2,959,282 | 738,528 | 4,461,896 | 0.18 |
| Eritrea | 4,597 | 3,006 | 2,214 | 9,818 | 0.24 | 370,490 | 158,531 | 146,811 | 675,832 | 0.23 |
| Spain | 489 | 39,831 | 3,304 | 43,623 | 0.11 | 33,697 | 1,186,826 | 239,597 | 1,460,120 | 0.12 |
| Estonia | 11 | 1,756 | 181 | 1,948 | 0.13 | 1,117 | 52,522 | 10,376 | 64,014 | 0.13 |
| Ethiopia | 82,032 | 38,510 | 31,007 | 151,549 | 0.22 | 6,812,739 | 2,061,119 | 2,149,253 | 11,023,112 | 0.23 |
| Finland | 45 | 4,821 | 709 | 5,574 | 0.11 | 3,011 | 156,131 | 37,499 | 196,642 | 0.12 |
| Fiji | 72 | 624 | 222 | 918 | 0.15 | 10,849 | 28,804 | 13,721 | 53,374 | 0.17 |
| France | 1,115 | 51,203 | 8,800 | 61,117 | 0.11 | 50,084 | 1,774,803 | 418,744 | 2,243,630 | 0.13 |
| Micronesia (Federated States of) | 24 | 43 | 26 | 92 | 0.14 | 1,792 | 2,243 | 1,645 | 5,680 | 0.16 |
| Gabon | 1,603 | 716 | 483 | 2,803 | 0.19 | 115,511 | 35,942 | 32,811 | 184,264 | 0.20 |
| United Kingdom | 683 | 59,412 | 4,714 | 64,808 | 0.12 | 47,128 | 1,967,831 | 343,436 | 2,358,394 | 0.13 |
| Georgia | 172 | 11,660 | 530 | 12,362 | 0.25 | 17,838 | 295,626 | 38,740 | 352,203 | 0.21 |
| Ghana | 22,146 | 17,886 | 7,306 | 47,337 | 0.23 | 1,967,003 | 778,311 | 511,685 | 3,256,999 | 0.23 |
| Guinea | 16,421 | 7,712 | 4,614 | 28,747 | 0.24 | 1,446,172 | 369,868 | 335,575 | 2,151,614 | 0.25 |
| Gambia | 1,894 | 911 | 636 | 3,441 | 0.23 | 163,708 | 46,522 | 46,509 | 256,740 | 0.23 |
| Guinea-Bissau | 2,940 | 1,208 | 721 | 4,870 | 0.24 | 250,567 | 53,861 | 51,333 | 355,761 | 0.25 |
| Equatorial Guinea | 1,153 | 561 | 391 | 2,106 | 0.24 | 90,768 | 26,358 | 27,660 | 144,786 | 0.23 |
| Greece | 67 | 16,316 | 1,103 | 17,486 | 0.16 | 6,982 | 427,755 | 75,867 | 510,604 | 0.15 |
| Grenada | 5 | 83 | 17 | 106 | 0.14 | 544 | 3,174 | 1,456 | 5,174 | 0.16 |
| Guatemala | 3,679 | 6,411 | 3,822 | 13,913 | 0.17 | 367,942 | 305,997 | 253,298 | 927,237 | 0.18 |
| Guyana | 197 | 615 | 317 | 1,130 | 0.18 | 15,544 | 27,718 | 21,830 | 65,093 | 0.19 |
| Honduras | 1,242 | 4,209 | 1,722 | 7,173 | 0.20 | 114,939 | 202,951 | 107,512 | 425,402 | 0.19 |
| Croatia | 32 | 7,144 | 623 | 7,799 | 0.16 | 3,070 | 199,861 | 34,175 | 237,106 | 0.15 |
| Haiti | 7,780 | 10,065 | 2,973 | 20,818 | 0.23 | 619,441 | 392,961 | 326,393 | 1,338,795 | 0.23 |
| Hungary | 90 | 20,621 | 1,507 | 22,218 | 0.17 | 8,076 | 581,764 | 87,976 | 677,815 | 0.16 |
| Indonesia | 57,012 | 247,738 | 45,123 | 349,872 | 0.23 | 4,276,979 | 8,981,207 | 2,904,884 | 16,163,070 | 0.21 |
| India | 655,338 | 1,763,813 | 492,519 | 2,911,670 | 0.30 | 44,137,785 | 61,680,765 | 27,799,841 | 133,618,390 | 0.25 |
| Ireland | 66 | 2,944 | 390 | 3,401 | 0.13 | 4,147 | 110,501 | 28,023 | 142,671 | 0.14 |
| Iran (Islamic Republic of) | 4,624 | 59,186 | 23,492 | 87,302 | 0.22 | 419,379 | 2,380,725 | 1,444,674 | 4,244,777 | 0.20 |
| Iraq | 5,271 | 20,119 | 9,167 | 34,557 | 0.21 | 501,807 | 790,749 | 579,730 | 1,872,286 | 0.18 |
| Iceland | 2 | 160 | 24 | 186 | 0.09 | 180 | 6,578 | 1,599 | 8,357 | 0.12 |
| Israel | 164 | 4,210 | 421 | 4,794 | 0.12 | 7,153 | 151,803 | 31,948 | 190,905 | 0.13 |
| Italy | 593 | 69,087 | 5,760 | 75,439 | 0.13 | 37,310 | 1,832,677 | 342,210 | 2,212,198 | 0.13 |
| Jamaica | 191 | 2,097 | 426 | 2,714 | 0.14 | 25,353 | 77,396 | 40,954 | 143,703 | 0.16 |
| Jordan | 417 | 3,181 | 1,176 | 4,774 | 0.18 | 45,966 | 154,406 | 81,492 | 281,865 | 0.17 |
| Japan | 2,378 | 109,401 | 19,500 | 131,278 | 0.11 | 89,885 | 3,370,373 | 761,986 | 4,222,245 | 0.13 |
| Kazakhstan | 981 | 24,996 | 5,288 | 31,265 | 0.20 | 90,782 | 837,692 | 312,305 | 1,240,779 | 0.19 |
| Kenya | 46,060 | 18,122 | 16,541 | 80,724 | 0.22 | 3,757,673 | 974,136 | 1,171,683 | 5,903,492 | 0.22 |
| Kyrgyzstan | 610 | 7,102 | 1,034 | 8,747 | 0.24 | 59,151 | 243,264 | 73,876 | 376,291 | 0.20 |
| Cambodia | 5,841 | 11,054 | 4,117 | 21,012 | 0.25 | 458,948 | 507,879 | 296,734 | 1,263,561 | 0.22 |
| Kiribati | 32 | 58 | 31 | 121 | 0.15 | 3,657 | 2,610 | 1,858 | 8,126 | 0.18 |
| Saint Kitts and Nevis | 2 | 27 | 9 | 37 | 0.08 | 157 | 1,008 | 646 | 1,810 | 0.10 |
| Republic of Korea | 1,018 | 32,678 | 4,263 | 37,959 | 0.14 | 51,243 | 1,209,735 | 221,886 | 1,482,863 | 0.14 |
| Kuwait | 74 | 900 | 255 | 1,229 | 0.20 | 6,553 | 57,468 | 22,513 | 86,534 | 0.16 |
| Lao People's Democratic Republic | 7,139 | 5,948 | 1,823 | 14,911 | 0.32 | 566,244 | 232,728 | 128,322 | 927,293 | 0.31 |
| Lebanon | 94 | 3,232 | 639 | 3,964 | 0.19 | 10,465 | 112,845 | 34,692 | 158,002 | 0.16 |
| Liberia | 4,373 | 2,656 | 1,243 | 8,272 | 0.24 | 396,363 | 124,917 | 88,010 | 609,290 | 0.25 |
| Libya | 300 | 3,571 | 1,135 | 5,006 | 0.19 | 29,378 | 152,917 | 69,273 | 251,568 | 0.17 |
| Saint Lucia | 7 | 149 | 34 | 190 | 0.15 | 736 | 5,628 | 3,959 | 10,324 | 0.17 |
| Sri Lanka | 1,591 | 26,274 | 7,050 | 34,915 | 0.25 | 119,587 | 879,575 | 338,468 | 1,337,630 | 0.22 |
| Lesotho | 2,517 | 1,187 | 1,048 | 4,752 | 0.16 | 169,306 | 55,871 | 67,964 | 293,141 | 0.16 |
| Lithuania | 59 | 4,633 | 670 | 5,362 | 0.15 | 4,258 | 136,747 | 35,863 | 176,868 | 0.15 |
| Luxembourg | 6 | 347 | 55 | 409 | 0.11 | 315 | 13,237 | 3,042 | 16,594 | 0.13 |
| Latvia | 26 | 4,022 | 356 | 4,404 | 0.15 | 2,403 | 107,636 | 19,690 | 129,729 | 0.15 |
| Morocco | 3,966 | 22,244 | 6,066 | 32,276 | 0.16 | 343,072 | 960,826 | 434,726 | 1,738,624 | 0.16 |
| Monaco | 0 | 20 | 4 | 24 | 0.08 | 27 | 691 | 244 | 962 | 0.10 |
| Republic of Moldova | 147 | 7,797 | 1,029 | 8,973 | 0.21 | 12,745 | 216,420 | 54,754 | 283,919 | 0.19 |
| Madagascar | 18,499 | 15,908 | 7,438 | 41,845 | 0.26 | 1,636,597 | 736,620 | 526,020 | 2,899,238 | 0.26 |
| Maldives | 16 | 108 | 40 | 164 | 0.13 | 1,471 | 5,629 | 2,482 | 9,583 | 0.14 |
| Mexico | 5,845 | 55,046 | 22,691 | 83,582 | 0.14 | 703,897 | 2,323,377 | 1,369,101 | 4,396,375 | 0.15 |
| Marshall Islands | 21 | 24 | 9 | 54 | 0.15 | 1,850 | 1,247 | 604 | 3,701 | 0.19 |
| The former Yugoslav Republic of Macedonia | 24 | 3,199 | 176 | 3,399 | 0.17 | 2,950 | 100,653 | 15,362 | 118,965 | 0.17 |
| Mali | 27,931 | 11,128 | 6,891 | 45,949 | 0.27 | 2,497,069 | 466,214 | 508,379 | 3,471,662 | 0.27 |
| Malta | 2 | 446 | 23 | 471 | 0.16 | 249 | 14,403 | 2,121 | 16,772 | 0.15 |
| Myanmar | 21,584 | 69,244 | 18,407 | 109,235 | 0.25 | 1,652,908 | 2,511,951 | 1,106,407 | 5,271,265 | 0.23 |
| Montenegro | 7 | 1,006 | 65 | 1,077 | 0.19 | 843 | 31,253 | 5,078 | 37,174 | 0.18 |
| Mongolia | 395 | 3,954 | 825 | 5,173 | 0.27 | 36,154 | 146,490 | 56,074 | 238,718 | 0.24 |
| Mozambique | 42,371 | 12,524 | 15,597 | 70,492 | 0.23 | 3,393,769 | 652,875 | 1,018,782 | 5,065,426 | 0.24 |
| Mauritania | 3,992 | 1,858 | 1,106 | 6,955 | 0.23 | 328,441 | 92,532 | 77,378 | 498,352 | 0.23 |
| Mauritius | 77 | 722 | 229 | 1,027 | 0.11 | 5,365 | 32,512 | 13,970 | 51,847 | 0.13 |
| Malawi | 18,688 | 9,165 | 5,213 | 33,066 | 0.22 | 1,525,775 | 417,365 | 350,786 | 2,293,926 | 0.22 |
| Malaysia | 1,456 | 17,740 | 6,745 | 25,941 | 0.18 | 303,150 | 746,223 | 377,679 | 1,427,051 | 0.19 |
| Namibia | 991 | 1,183 | 556 | 2,730 | 0.19 | 73,400 | 54,444 | 36,551 | 164,394 | 0.18 |
| Niger | 34,101 | 9,144 | 7,020 | 50,265 | 0.28 | 2,980,654 | 447,350 | 535,383 | 3,963,387 | 0.28 |
| Nigeria | 312,269 | 87,248 | 98,218 | 497,736 | 0.24 | 27,395,177 | 4,538,530 | 7,154,007 | 39,087,714 | 0.25 |
| Nicaragua | 692 | 3,958 | 1,205 | 5,855 | 0.20 | 69,801 | 172,371 | 74,475 | 316,647 | 0.19 |
| Niue | 0 | 1 | 0 | 1 | 0.12 | 20 | 23 | 23 | 67 | 0.15 |
| Netherlands | 185 | 14,655 | 1,288 | 16,128 | 0.12 | 10,923 | 483,521 | 81,299 | 575,743 | 0.13 |
| Norway | 89 | 3,306 | 515 | 3,909 | 0.09 | 3,500 | 129,441 | 26,341 | 159,282 | 0.12 |
| Nepal | 9,829 | 28,508 | 8,350 | 46,687 | 0.25 | 762,892 | 1,099,488 | 507,066 | 2,369,446 | 0.23 |
| Nauru | 1 | 2 | 1 | 4 | 0.12 | 145 | 126 | 81 | 352 | 0.14 |
| New Zealand | 55 | 2,713 | 369 | 3,137 | 0.11 | 3,143 | 106,523 | 24,449 | 134,115 | 0.13 |
| Oman | 80 | 969 | 553 | 1,601 | 0.17 | 7,913 | 54,457 | 38,407 | 100,776 | 0.15 |
| Pakistan | 117,630 | 151,153 | 62,399 | 331,181 | 0.25 | 9,569,515 | 5,744,589 | 4,154,295 | 19,468,399 | 0.23 |
| Panama | 433 | 1,578 | 718 | 2,729 | 0.14 | 31,055 | 81,695 | 43,565 | 156,316 | 0.15 |
| Peru | 1,800 | 14,027 | 5,296 | 21,123 | 0.16 | 193,441 | 714,504 | 325,454 | 1,233,399 | 0.17 |
| Philippines | 22,391 | 84,891 | 16,177 | 123,459 | 0.22 | 2,115,692 | 3,733,072 | 1,175,372 | 7,024,136 | 0.21 |
| Palau | 2 | 8 | 5 | 14 | 0.12 | 224 | 354 | 291 | 869 | 0.14 |
| Papua New Guinea | 4,495 | 3,247 | 2,615 | 10,356 | 0.19 | 391,337 | 186,642 | 171,289 | 749,268 | 0.21 |
| Poland | 292 | 53,628 | 5,300 | 59,220 | 0.16 | 29,937 | 1,634,361 | 342,440 | 2,006,738 | 0.15 |
| Democratic People's Republic of Korea | 2,821 | 58,899 | 8,726 | 70,446 | 0.31 | 251,665 | 1,717,841 | 485,192 | 2,454,698 | 0.27 |
| Portugal | 172 | 9,683 | 834 | 10,689 | 0.11 | 8,476 | 328,946 | 60,446 | 397,869 | 0.13 |
| Paraguay | 544 | 4,333 | 1,460 | 6,337 | 0.20 | 57,423 | 190,058 | 91,274 | 338,755 | 0.18 |
| Qatar | 22 | 330 | 202 | 553 | 0.20 | 2,694 | 34,173 | 16,831 | 53,698 | 0.16 |
| Romania | 750 | 43,330 | 3,245 | 47,325 | 0.19 | 61,780 | 1,212,430 | 229,867 | 1,504,076 | 0.18 |
| Russian Federation | 5,005 | 296,462 | 48,344 | 349,811 | 0.17 | 355,466 | 8,917,419 | 2,503,254 | 11,776,140 | 0.16 |
| Rwanda | 9,148 | 5,586 | 4,042 | 18,777 | 0.24 | 799,620 | 312,610 | 287,947 | 1,400,176 | 0.24 |
| Saudi Arabia | 725 | 14,242 | 3,425 | 18,393 | 0.20 | 63,972 | 621,656 | 245,271 | 930,899 | 0.16 |
| Sudan | 33,809 | 18,851 | 16,457 | 69,117 | 0.23 | 2,866,999 | 996,509 | 1,103,259 | 4,966,767 | 0.23 |
| Senegal | 11,730 | 5,442 | 3,697 | 20,869 | 0.21 | 943,350 | 286,096 | 246,998 | 1,476,444 | 0.22 |
| Singapore | 79 | 2,873 | 159 | 3,110 | 0.13 | 4,220 | 104,021 | 12,217 | 120,457 | 0.13 |
| Solomon Islands | 171 | 322 | 141 | 634 | 0.21 | 15,232 | 15,296 | 9,544 | 40,072 | 0.22 |
| Sierra Leone | 15,639 | 4,974 | 3,757 | 24,370 | 0.24 | 1,352,883 | 240,047 | 277,807 | 1,870,737 | 0.25 |
| El Salvador | 406 | 4,320 | 2,345 | 7,071 | 0.17 | 48,046 | 178,442 | 130,906 | 357,394 | 0.17 |
| San Marino | 0 | 18 | 4 | 22 | 0.07 | 19 | 564 | 196 | 778 | 0.09 |
| Somalia | 23,833 | 4,847 | 5,516 | 34,196 | 0.24 | 1,987,711 | 266,009 | 408,945 | 2,662,666 | 0.25 |
| Serbia | 139 | 16,520 | 860 | 17,519 | 0.15 | 12,007 | 514,804 | 74,772 | 601,583 | 0.16 |
| South Sudan | 16,421 | 5,671 | 5,390 | 27,482 | 0.22 | 1,352,267 | 263,177 | 373,911 | 1,989,355 | 0.22 |
| Sao Tome and Principe | 105 | 74 | 52 | 231 | 0.19 | 8,820 | 4,067 | 3,673 | 16,560 | 0.20 |
| Suriname | 43 | 225 | 113 | 381 | 0.16 | 3,376 | 12,043 | 9,529 | 24,948 | 0.16 |
| Slovakia | 49 | 7,558 | 628 | 8,234 | 0.16 | 4,352 | 228,710 | 44,934 | 277,996 | 0.15 |
| Slovenia | 12 | 2,131 | 356 | 2,499 | 0.13 | 1,390 | 69,823 | 17,391 | 88,603 | 0.14 |
| Sweden | 236 | 7,473 | 965 | 8,675 | 0.10 | 7,027 | 252,070 | 52,417 | 311,513 | 0.12 |
| Swaziland | 1,277 | 590 | 521 | 2,388 | 0.17 | 97,958 | 31,407 | 35,647 | 165,012 | 0.17 |
| Seychelles | 13 | 42 | 31 | 86 | 0.14 | 940 | 2,002 | 1,371 | 4,313 | 0.16 |
| Syrian Arab Republic | 1,242 | 13,440 | 2,354 | 17,036 | 0.13 | 138,178 | 528,957 | 179,282 | 846,416 | 0.10 |
| Chad | 32,543 | 6,927 | 5,835 | 45,305 | 0.26 | 2,776,446 | 338,234 | 437,822 | 3,552,503 | 0.27 |
| Togo | 9,134 | 3,826 | 2,487 | 15,447 | 0.24 | 788,672 | 201,241 | 184,427 | 1,174,340 | 0.24 |
| Thailand | 5,659 | 66,240 | 21,924 | 93,823 | 0.19 | 394,699 | 2,458,498 | 1,088,011 | 3,941,207 | 0.18 |
| Tajikistan | 2,499 | 7,132 | 1,504 | 11,135 | 0.23 | 227,791 | 270,967 | 122,886 | 621,643 | 0.20 |
| Turkmenistan | 956 | 8,931 | 1,528 | 11,415 | 0.25 | 83,870 | 335,219 | 107,461 | 526,551 | 0.22 |
| Timor-Leste | 692 | 651 | 296 | 1,638 | 0.23 | 59,196 | 28,480 | 21,968 | 109,644 | 0.22 |
| Tonga | 10 | 63 | 19 | 92 | 0.13 | 811 | 2,680 | 1,076 | 4,567 | 0.15 |
| Trinidad and Tobago | 47 | 986 | 286 | 1,319 | 0.10 | 3,409 | 40,514 | 20,127 | 64,050 | 0.12 |
| Tunisia | 450 | 8,736 | 1,766 | 10,952 | 0.18 | 42,455 | 330,387 | 114,440 | 487,282 | 0.17 |
| Turkey | 2,248 | 65,929 | 7,343 | 75,520 | 0.18 | 234,077 | 2,533,982 | 587,534 | 3,355,593 | 0.16 |
| Tuvalu | 2 | 6 | 3 | 11 | 0.13 | 176 | 233 | 180 | 589 | 0.15 |
| United Republic of Tanzania | 48,814 | 20,180 | 21,216 | 90,210 | 0.22 | 3,919,608 | 1,151,779 | 1,407,384 | 6,478,771 | 0.23 |
| Uganda | 44,407 | 17,422 | 20,469 | 82,298 | 0.23 | 3,696,370 | 923,440 | 1,413,613 | 6,033,424 | 0.24 |
| Ukraine | 1,966 | 121,104 | 12,228 | 135,298 | 0.20 | 139,801 | 3,151,315 | 683,667 | 3,974,783 | 0.18 |
| Uruguay | 59 | 2,588 | 472 | 3,119 | 0.10 | 5,226 | 92,178 | 24,866 | 122,269 | 0.12 |
| United States of America | 5,726 | 236,052 | 40,732 | 282,510 | 0.11 | 292,884 | 8,793,415 | 2,042,633 | 11,128,932 | 0.12 |
| Uzbekistan | 3,399 | 35,865 | 4,398 | 43,662 | 0.24 | 323,063 | 1,261,612 | 351,320 | 1,935,994 | 0.20 |
| Saint Vincent and the Grenadines | 7 | 80 | 20 | 108 | 0.15 | 675 | 3,233 | 1,717 | 5,625 | 0.16 |
| Venezuela (Bolivarian Republic of) | 2,038 | 14,637 | 9,226 | 25,902 | 0.18 | 206,225 | 730,540 | 580,829 | 1,517,594 | 0.18 |
| Viet Nam | 10,706 | 94,987 | 23,578 | 129,271 | 0.25 | 1,146,087 | 3,278,458 | 1,323,610 | 5,748,155 | 0.23 |
| Vanuatu | 42 | 154 | 45 | 241 | 0.21 | 4,009 | 6,975 | 2,716 | 13,699 | 0.20 |
| Samoa | 21 | 99 | 34 | 154 | 0.15 | 1,775 | 4,442 | 2,016 | 8,233 | 0.17 |
| Yemen | 9,940 | 14,405 | 6,972 | 31,317 | 0.19 | 957,469 | 681,099 | 524,049 | 2,162,616 | 0.19 |
| South Africa | 42,071 | 35,595 | 19,742 | 97,408 | 0.16 | 2,649,414 | 1,495,013 | 1,139,386 | 5,283,813 | 0.16 |
| Zambia | 19,892 | 6,188 | 7,156 | 33,235 | 0.23 | 1,671,080 | 321,571 | 492,447 | 2,485,097 | 0.23 |
| Zimbabwe | 13,869 | 7,767 | 4,759 | 26,394 | 0.19 | 1,131,375 | 346,491 | 353,924 | 1,831,790 | 0.19 |
| **Gobal** | **2,503,679** | **8,170,727** | **1,950,088** | **12,624,495** | **0.23** | **201,721,743** | **276,224,287** | **118,466,141** | **596,412,171** | **0.22** |

values in this table show absolute number of deaths and DALYs, PAF: population attributable fraction

A5: Links between environmental risks and the Sustainable Development Goals (SDGs)

The main interactions between environmental risks and the SDGs (Table A3) include: traditional risks such as unsafe water and sanitation, and use of solid fuels for cooking with Goals 1, 5, 6 and 7; less traditional risks such as ambient air pollution with Goals 7, 9, 11 and 12; issues of inequality – given that certain environmental risks disproportionally affect certain population subgroups – with Goals 1, 5 and 10; use of chemicals with agriculture, occupation and the industry, and thus Goals 2, 8 and 9; emerging risks such as climate change and conservation of biodiversity and natural resources, and thus Goals 13, 14 and 15; education, given the link between school attendance and absence of sanitation services, or time lost to fetching wood or water in competition with education, and therefore Goal 4. Finally, Goal 3 – ensure healthy lives and promote well-being can be attained through environmental action, as close to one quarter of the global disease burden could be prevented through healthier environments.

Table A3: List of the 17 Sustainable Development Goals

| Goal 1: End poverty in all its forms everywhere |
| --- |
| Goal 2: End hunger, achieve food security and improved nutrition and promote sustainable agriculture |
| Goal 3: Ensure healthy lives and promote well-being for all at all ages |
| Goal 4: Ensure inclusive and equitable quality education and promote lifelong learning opportunities for all |
| Goal 5: Achieve gender equality and empower all women and girls |
| Goal 6: Ensure availability and sustainable management of water and sanitation for all |
| Goal 7: Ensure access to affordable, reliable, sustainable and modern energy for all |
| Goal 8: Promote sustained, inclusive and sustainable economic growth, full and productive employment and decent work for all |
| Goal 9: Build resilient infrastructure, promote inclusive and sustainable industrialization and foster innovation |
| Goal 10: Reduce inequality within and among countries |
| Goal 11: Make cities and human settlements inclusive, safe, resilient and sustainable |
| Goal 12: Ensure sustainable consumption and production patterns |
| Goal 13: Take urgent action to combat climate change and its impacts |
| Goal 14: Conserve and sustainably use the oceans, seas and marine resources for sustainable development |
| Goal 15: Protect, restore and promote sustainable use of terrestrial ecosystems, sustainably manage forests, combat desertification, and halt and reverse land degradation and halt biodiversity loss |
| Goal 16: Promote peaceful and inclusive societies for sustainable development, provide access to justice for all and build effective, accountable and inclusive institutions at all levels |
| Goal 17: Strengthen the means of implementation and revitalize the global partnership for sustainable development |

**References**

1. World Health Organization. Global Health Observatory (GHO) data [Internet]. WHO. undated [cited 2015 Jul 16]. Available from: http://www.who.int/gho/en/

2. Smith KR, Corvalán CF, Kjellstrom T. How much global ill health is attributable to environmental factors? Epidemiology. 1999;10(5):573–84.

1. Although natural UV radiation from space is not modifiable (or only in a limited way, such as by reducing substances that destroy the ozone layer), individual behaviour to protect oneself against UV radiation is modifiable. UV are therefore included in our assessment of the environmental disease burden. [↑](#footnote-ref-1)
